# Migrant healthcare access in Morocco: A narrative review

**DOI:** 10.4102/jphia.v16i1.806

**Published:** 2025-02-21

**Authors:** Hicham El Bouri, Adil Najdi

**Affiliations:** 1 Laboratory of Epidemiology and Public Health, Faculty of Medicine and Pharmacy of Tangier, Abdelmalek Essaadi University, Tangier, Morocco

**Keywords:** migrant, healthcare access, narrative review, Morocco, barrier

## Abstract

**Background:**

Morocco is at the forefront of a complex migratory dynamic because of its strategic geographical position, situated at the crossroads between sub-Saharan Africa, Europe and the Middle East, which has shifted migratory flows in the kingdom. As a result, it must meet migrants’ needs, especially in healthcare. Although several studies have explored aspects of healthcare access for migrant populations, there is a notable lack of comprehensive reviews synthesising this research. This gap hinders a holistic understanding of the challenges faced by migrants in accessing healthcare and its implications for public health policy.

**Aim:**

This study aimed to analyse and integrate existing research findings to offer decision-makers evidence-based insights for developing informed and effective strategies.

**Setting:**

The study examined healthcare access for migrants within the Moroccan healthcare system.

**Method:**

We employed a narrative review method consisting of three key steps: defining the research question, designing the search strategy and selecting articles based on predefined criteria. Seventeen documents were ultimately included in this exploratory review.

**Results:**

Reviewed studies identified five main obstacles hindering migrants’ access to Morocco’s healthcare system: legal restrictions, procedural requirements, high healthcare costs, lack of knowledge about rights and the Moroccan health system and fear of being reported to immigration authorities, particularly among irregular migrants.

**Conclusion:**

The study identified significant gaps in the existing research, particularly the lack of data stratification by socio-cultural and administrative criteria. Additionally, there is limited investigation into how these barriers affect both physical and mental health. The analysis also revealed a scarcity of mixed-method studies that could provide a more comprehensive understanding of these issues.

**Contribution:**

Addressing these gaps would enable decision-makers to better understand the underlying issues affecting healthcare access for migrants in Morocco, ultimately leading to the development of strategies to improve access.

## Introduction

The Kingdom of Morocco occupies a pivotal position within a complex migratory landscape. Strategically situated at the crossroads of Africa, Europe and the Arab world, Morocco has transitioned from a transit country to a host country over the past decades.^1^ This shift reflects evolving migratory flows, driven by geopolitical instability, economic disparities and the tightening of border controls in neighbouring regions.^2^ As a result, Morocco now faces the dual challenge of integrating diverse migrant populations and addressing their pressing needs, particularly in healthcare, a cornerstone of human rights and social stability.^3^

Access to healthcare for migrant populations is a multifaceted issue influenced by legal, administrative, social and cultural barriers. In Morocco, these challenges are compounded by limited healthcare infrastructure, inconsistent policy frameworks and a lack of standardised practices to address the specific needs of migrants.^4^ For example, legal requirements such as proof of residence or identity documents often exclude undocumented migrants from accessing essential healthcare services. Additionally, language barriers and cultural differences can hinder communication between migrants and healthcare providers, further limiting the quality of care delivered.^1^

Globally, migrant access to healthcare varies widely. In inclusive systems like those in Spain and Italy, migrants benefit from progressive policies facilitating access to basic and emergency care.^5^ Conversely, restrictive systems, such as those in some transitory countries like Libya, leave migrants vulnerable to exploitation, political instability and inadequate health services.^6^ These international examples provide valuable comparative insights, highlighting both best practices and critical pitfalls. They also emphasise the urgent need for Morocco to develop a context-specific approach that ensures equitable healthcare access for all, regardless of legal or residency status.

Despite a growing interest from researchers, governmental bodies and non-governmental organisations in improving healthcare access for migrants, significant gaps remain in our understanding of this issue within Morocco. Existing studies often lack comprehensive data and fail to capture the nuanced socio-cultural and administrative barriers migrants face. This knowledge gap hampers the development of informed policies and interventions tailored to Morocco’s unique context.

This narrative review aims to address these gaps by synthesising existing literature on migrant healthcare access in Morocco. By identifying key barriers, highlighting grey areas in the research, and drawing comparisons with international contexts, this review seeks to provide decision-makers with evidence-based insights. Ultimately, this work aspires to inform public health strategies that not only address immediate challenges but also pave the way for sustainable solutions to ensure equitable healthcare for migrant populations in Morocco.

## Methods

In order to meet our objective, we used a narrative review method that consists of three steps. The first one was to identify the research question which was: ‘*What are the main barriers for health care access for migrants in Morocco*?’

Then, we designed a search strategy that was applied to various literature databases such as MEDLINE, Google Scholar, Scopus and so on. The search was conducted in February 2023 and focused on articles published between January 2012 and December 2022, without language restriction, using a search strategy combining the following keywords (or their synonyms): ‘Migrant’, ‘Healthcare access’, ‘Morocco’. The term ‘migrant’ is used generically in this review and includes regular migrants, irregular migrants, refugees and asylum seekers. A grey literature review was also conducted by performing either a thematic search from institutional websites or a keyword search from traditional search engines. The keywords used are the same as those used for the review of the published literature.

In the third step, we selected articles based on defined inclusion and exclusion criteria. The inclusion criteria focused on primary or secondary research that explicitly addressed access to healthcare for international migrants, irrespective of their legal or residency status. This included studies examining barriers, facilitators or interventions related to healthcare access for migrant populations. Conversely, the exclusion criteria ruled out studies that exclusively focused on internal migration within a single country, as these do not align with the scope of this review, which emphasises international migratory movements.

### Ethical considerations

This article followed all ethical standards for research without direct contact with human or animal subjects.

## Results

Seventeen documents were selected in this exploratory narrative review. Among the 17 publications included in this narrative review, 9 (53%) were reports from intergovernmental organisations, research institutes or reports from non-governmental organisations (non-governmental organisations), 4 (23.5%) were Master’s theses, 3 (17.6%) were original articles and one (5.9%) publication was a synthesis article. Regarding the methodology used in the selected articles, 6 (35.3%) publications have not specified the method adopted, 4 (23.5%) adopted a quantitative approach (cross sectional), 4 (23.5%) were qualitative studies (interview and observation), 2 (11.8%) were synthesis papers and 1 (5.9%) publication adopted a mixed approach (Table 1). Given the exploratory nature of this review, we have not examined quality criteria (Table 1). We involved compiling, summarising and reporting the following information from all selected articles: title of the publication, author’s name, type of publication, year of publication, type of study and target population.

**TABLE 1:** Overview of documents included in this review.

Title of the publication	Author’s name	Type of publication	Year of publication	Type of study	Target population
Accès des réfugiés et migrants aux soins de santé du secteur public, Rabat^7^	Chaoui El Yousfi A.	Master thesis	2018	Cross sectional	All profiles
Violences, Vulnérabilité et Migration: Bloqués aux Portes de l’Europe^8^	Médecins Sans frontières	Report	2013	Not specified	All profiles
Sexual violence and sub-Saharan migrants in Morocco: A community-based participatory assessment using respondent driven sampling^9^	Keygnaert I & Al	Original article	2014	Qualitative	Irregular migrants
Health and Sub-Saharan Migrants in Morocco: Recent Changes and Future Trends in Healthcare Access and Immigration Policy^10^	Doppelt S.	Original article	2013	Not specified	All profiles
Cartographie des acteurs intervenant dans le domaine de la prévention combinée auprès des migrants au Maroc^11^	Directorate of Epidemiology and Disease Control	Report	2013	Qualitative	All profiles
Prevalence and determinants of intercourse without condoms among migrants and refugees in Morocco, 2021: a cross-sectional study^12^	Essayagh T.	Original article	2021	Cross sectional	All profiles
Axes prioritaires de la plateforme nationale de protection des migrants pour la protection de l’enfance migrante au Maroc^13^	NMPP	Report	2016	Not specified	All profiles
Les déterminants socioculturels d’accès à la santé des personnes migrantes au Maroc^14^	Economia, HEM Research Center	Report	2020	Qualitative	All profiles
L’accès aux soins des migrants subsahariens au Maroc : une analyse de la situation dans le cadre de la mise en œuvre PSRSI de Casablanca-Settat^15^	Boughnisa A.	Master thesis	2018	Qualitative	All profiles
Besoins et attentes des adolescents et des jeunes migrants en santé de la reproduction^16^	Mara D.	Master thesis	2019	Not specified	All profiles
La lutte contre le sida au Maroc: opportunités et limites pour l’accès aux soins des personnes en migration^17^	Fidelin C.	Review article	2021	Review	All profiles
Favoriser la santé et la protection des migrant(e)s en situation de vulnérabilité au Maroc, Tunisie, Libye, Egypte, Yémen et Soudan^18^	IOM	Report	2022	Not specified	All profiles
La migration forcée au Maroc: Résultats de l’Enquête Nationale de 2021 Septembre^2^	High Commission for Planning	Report	2021	Cross sectional	All profiles
HIV integrated behavioral and biological surveillance surveys among sub-Saharan migrants in an irregular administrative situation in Morocco^19^	Directorate of Epidemiology and Disease Control	Report	2013	Cross sectional	Irregular migrants
Promotion des services Psychosociaux et de services d’assistance aux migrants aux Maroc^20^	IOM	Report	2017	Mixed-methods approach, quantitative and qualitative	All profiles
Promotion de la santé des migrants en situation irrégulière dans la région de l’Oriental au Maroc^21^	El Bouri H.	Master thesis	2016	Review	All profiles
Plan stratégique national santé et immigration 2021–2025^22^	Ministry of Health and Social Protection	Report	2020	Not specified	All profiles

PSRSI, Plan Stratégique National Santé et Immigration; HEM, Institut des Hautes Etudes en Management; IOM, International Organization for Migration.

Studies reviewed identified five main obstacles that make it difficult or impede migrants’ enjoyment of their right to health, including:

- Legal restrictions on access to healthcare services (*n* = 8).
- Procedural requirements for accessing to healthcare services that migrants cannot always meet (*n* = 7).
- High costs of healthcare (*n* = 8).
- Lack of knowledge about the rights and the functioning of the Moroccan health system because of insufficient communication (*n* = 10).
- The fear of irregular migrants of being reported to immigration authorities (*n* = 6).

### Legal restrictions on access to healthcare services

Regardless of their legal status, migrants have free access to all healthcare services offered by the primary healthcare (PHC) services network.^7,8,9,10^ Free access is also granted for curative and preventive services within the sexual and reproductive health (SRH) programme as well as programmes to fight tuberculosis, malaria and acquired immunodeficiency syndrome (AIDS).^7,11,12^ Access to PHC services is usually provided in big cities. This is to raise awareness among health professionals about the vulnerabilities of the migrant population. However, in Moroccan cities where civil society is less mobilised and healthcare facilities are less accustomed to receiving migrants, access to healthcare may be more difficult from the primary level of care, even for children.^13,14^ However, access to secondary and tertiary care is still difficult, if not impossible, for many migrants because of the lack of medical coverage even in the case of refugees who are protected by their status and supported by several associations.^14^ Elsewhere, regular migrants have access to all healthcare facilities in the same way as Moroccans as long as they have full medical coverage for those declared by their employers to the social security system.^14^

### Procedural requirements

Many irregular migrants have no documents that prove their usual address to attend a PHC centre.^8,14,15^ In the case of migrants who can afford to rent a room in a flat, they are denied proof of residence by landlords.^14^ This is further complicated by the fact that the migrant population is highly mobile and there is difficulty proving where they live.^14^ Also, many migrants are unable to provide an ID card to be admitted into a secondary or tertiary healthcare facility level.^10,16,17,18^ This has led to refusals of care, especially at university hospitals.^15^ In this sense, migrant support associations are active in facilitating access to healthcare facilities, because most of the information required is often recorded by the community worker attending to the migrant.^14,15^

### Costs of healthcare

As mentioned earlier in the text, migrants benefit from free services at the PHC level. However, most of the healthcare centres visited by the migrant population lack medicines because of the staffing system, which does not consider irregular migrants as they are not registered as living in the neighbourhood.^7,8,11,12,14^ This results in a high rate of prescriptions that remain at the migrant’s expense. Also, financial difficulties emerge from the secondary level of care, mainly for migrants in an irregular situation, and also for those who do not have enough resources to afford specialised care or do not have health insurance.^2,13,14,15,^ In some cases where hospitals have agreed to admit migrants before they pay, the administration of these establishments’ resorts to illegal practices such as confiscating the patient’s identification (ID) card, or not issuing a birth certificate in the case of delivery until the cost of treatment has been recovered.^14,15^

### Lack of knowledge about the rights and the functioning of the Moroccan health system

Evidence collected in this review has shown that language and communication barriers prevent migrants not only from accessing information about their rights and the functioning of the Moroccan health system, but also from communicating effectively with administrative and medical staff. Migrants arriving in Morocco are of various nationalities. Cultural and language differences can hinder communication and/or prevent the understanding of certain medical information, especially regarding human immunodeficiency viruses (HIV) and AIDS (testing, care and education), SRH and mental health, or even being excluded from information channels.^11,16,17,19,20,21^ Also, a select number of migrants interviewed in several studies state that they were unaware of the Moroccan health system because of a lack of information on the healthcare pathways, including the existing healthcare services to which they can have access as well as their limit,^14,22^ and explains the misconception that they have no right to access any healthcare facility in Morocco.^7,11^ Migrants have blamed the lack of diligence of some government administrations in providing information to migrants as well as the lack of training of healthcare professionals in intercultural communication and their awareness on migrants’ needs.^14,15,22^ Raising awareness and spreading healthcare or health-related information are only carried out by a limited number of non-governmental organisations in Morocco.^14^

### Fear of being reported to immigration authorities

Irregular migrants stated that their administrative status plays a central role in accessing healthcare facilities in Morocco.^14,22^ Some of them expressed mistrust or fear towards Moroccan institutions.^14^ This was also confirmed by some healthcare professionals. Except for serious cases or high-risk pregnancies, most irregular migrants avoid going to a healthcare centre or a public hospital for fear of arrest, which could lead to forced displacement to another city or to being deported to the border, which would thwart their migration plans.^11,14,17,19^ This permanent fear is increased when migrants are in an area where there are no non-governmental organisations.^8^ In addition, several deaths among irregular migrants have been attributed to untreated illnesses because of fear of attending public healthcare facilities.^14^

## Discussion

This article has reviewed studies that highlight barriers to access to healthcare among the migrant population in Morocco.

The health-related experiences of migrants in Morocco are very heterogeneous and depend on a combination of factors, mainly the path of migration, country of origin, state of health at departure and arrival and residence status.^14^ This last characteristic seems to largely determine their access to healthcare in Morocco.

Regarding legislation, international human rights law sets out obligations that states are required to fulfil in relation to migrants, regardless of their status, by introducing measures to ensure access to a set of public services, including healthcare services.^23^ International literature shows that the access rights or care packages provided to migrants vary from one country to another.^24,25^ The spectrum of healthcare provided to migrants varies from emergency care to the secondary level.^26^ Coronavirus disease 2019 (COVID-19) pandemic prompted many countries to review their policy on healthcare entitlements for migrants either by opening up healthcare entitlements or by expanding the healthcare package offered to them.^27^ In Morocco, current legislation grants free access to all migrants, regardless of their administrative status, into the PHC network, which is at the heart of the Moroccan health system providing basic care and whose resources are allocated according to the needs of a given territory.^14^ However, given that several migrants are not registered in the lists of the healthcare centres to which they are supposed to belong, the allocation to these healthcare centres does not take these migrants into account and they inevitably find themselves short of medicines. However, access to secondary and tertiary healthcare facilities systematically requires an advance payment of fees, which is usually impossible for migrants who are not covered by a health insurance to meet (including refugees however protected by their status) given the highly difficult socio-economic conditions they face. Indeed, migrants aspire to secure conditions of daily life by giving priority to spending on food and housing while waiting for the fulfilment of their migratory project.

Official procedures required by national laws for accessing healthcare are one of the main barriers for migrants who are seeking to fulfil their right to health in the host country. In order to be admitted into a healthcare facility, migrants often have to provide documents (such as proof of identity and/or proof of residence), or meet conditions that they are not usually able to.^28^ Also, the burdensome reimbursement procedures of healthcare providers are a major challenge, making them unwilling to accept patients because of the difficulty or inability to be reimbursed.^28^ In Germany, the German section of the Caritas Association revealed that some seriously ill asylum seekers were not receiving necessary healthcare because of the fact that access to healthcare was dependent on the successful completion of the asylum procedure.^25,29^ In France, a non-governmental organisation in Paris noted that it found a very high rate of healthcare refusal for those receiving a state medical aid, even if it is prohibited by French law.^26^ Morocco is no exception to the rule as proof of residence is required for registration and treatment in a PHC facility, while ID card is required for admission into a secondary or tertiary healthcare facility.^7,14,15^ Without these documents, individuals may face denial of care.

Healthcare costs can be a major barrier to access to care for migrants. The costs of consultations, hospitalisation, complementary examinations and/or medication can be high, which migrants cannot afford.^28^ This can also be a significant barrier for healthcare providers as the recovery process can be long and arduous.^28^ As mentioned earlier in the text, secondary and tertiary care is subject to prepayment in Morocco. Given the impossibility to do so, migrants either turn to migrant aid associations to provide them direct medical assistance or finance all or part of the costs incurred, or abandon the care risking serious damage to their health.^14^

The lack of information on migrants’ health rights, both for users and providers, is a major obstacle to access to care. In general, migrants use two main channels that they consider to be relatively reliable to obtain the information they need about access to healthcare, including information about their rights as well as the health facilities that are likely to receive them.^28^ These channels are word of mouth and non-governmental organisations. However, word of mouth has its limits. Firstly, there is a specific category of migrants who are not easily accessible.^28^ Secondly, information transmitted by word of mouth is often wrong, as in Italy, where some migrants were afraid to go to the hospital because they had heard that doctors could report them to the police.^28^ In addition, both hospital administrators and health professionals may not be aware of migrants’ health legislation and rights. A non-governmental organisations in Paris noted that a hospital has asked migrants to pay fees for some services even if they benefit from the medical state aid.^28^ Data collected in this review show that navigating the Moroccan health system is cumbersome for many migrants and requires the support of community workers. Our review highlights the fact that language and communication barriers significantly hinder access to public healthcare services for migrants in Morocco.^15^ This finding aligns with existing literature from other transitory and host countries, such as Spain and Italy, where linguistic differences often exacerbate challenges in navigating healthcare systems and understanding medical advice. In the Moroccan context, these barriers are compounded by limited cultural competency training among healthcare providers, as observed similarly in countries like Greece, which also face significant migratory pressures.^15^ There is a certain unwillingness among migrants, especially irregular ones, to communicate with hospital administrative and medical staff. Non-governmental organisations are generally involved either in facilitating communication with them or in providing the information that migrants need.^14^

The fear of accessing healthcare that irregular migrants feel is an important barrier to their access to healthcare. This fear, which may or may not be well-founded, is cited more often in some countries than in others, especially in those where the issue of migration is a public debate.^28^ It is also one of the reasons why irregular migrants provide false information, seek non-governmental organisation services or abandon their care.^28^ Moroccan data are similar to those in the international literature, as migrants in an irregular situation in Morocco prefer, in some extreme cases, to abandon their care rather than trust Moroccan healthcare institutions so that their project to cross the Mediterranean does not fall through.

While difficulties of access to healthcare for migrants in Morocco have been identified and are the result of a combination of various factors such as legislative, procedural, financial and communication, it would be of interest as a way of further research to investigate the migrant’s population access to healthcare by *stratifying it according to some criteria which includes gender, nationality, administrative status,* ethnicity, culture or migrant profile (accompanied or unaccompanied children). Indeed, this aspect has not been sufficiently studied as this would allow one to better understand the possible differences in access to healthcare between these migrants’ profiles. So far, only one study has highlighted a distinction between English-speaking and French-speaking migrants regarding their access to HIV and/or AIDS services.^19^ Furthermore, it would be interesting to carry out studies that investigate the extent to which these barriers impact on their physical and mental health, considering the heterogeneity of the migrant population. There is also a paucity of studies that take a mixed approach. This method may be interesting for future research in order to look at the issue in its entirety and would help us understand the explanations of many issues related to access to healthcare for the migrant population in Morocco.

## Conclusion

The right to health is a fundamental right of every human being, regardless of culture, race, ethnicity, religion, skin colour or administrative status. Sustainable development goals 3.8 on universal coverage for all, and 10.7 on facilitating migration and mobility engage states to do their utmost to update their legislation, their health services as well as to put in place financial protection measures to the benefit of these vulnerable people. Morocco is fully committed to respecting its international commitments and has made considerable progress in recent years in improving access to healthcare for the migrant population living in its territory. However, from the reviewed literatures, there is a need to improve on, mainly in terms of governance, legislation and financing. It would be very wise for the Moroccan health authorities to think about a collaborative model between stakeholders (including governmental institutions, non-governmental organisations, international agencies, healthcare providers and community-based organisations) by pooling their data and resources in order to take another step forward in terms of access to healthcare for migrants in the kingdom.
